# Structured clinical reasoning prompt enhances LLM’s diagnostic capabilities in diagnosis please quiz cases

**DOI:** 10.1007/s11604-024-01712-2

**Published:** 2024-12-03

**Authors:** Yuki Sonoda, Ryo Kurokawa, Akifumi Hagiwara, Yusuke Asari, Takahiro Fukushima, Jun Kanzawa, Wataru Gonoi, Osamu Abe

**Affiliations:** 1 Department of Radiology, Graduate School of Medicine, The University of Tokyo, 7-3-1 Hongo, Bunkyo-Ku, Tokyo, 113-8655 Japan; 2 Department of Radiology, Juntendo University School of Medicine, 2-1-1, Hongo, Bunkyo-Ku, Tokyo, 111-8421 Japan

**Keywords:** Large language models, Artificial intelligence, Clinical reasoning, Claude 3.5 Sonnet, Radiology, Prompt engineering

## Abstract

**Purpose:**

Large Language Models (LLMs) show promise in medical diagnosis, but their performance varies with prompting. Recent studies suggest that modifying prompts may enhance diagnostic capabilities. This study aimed to test whether a prompting approach that aligns with general clinical reasoning methodology—specifically, using a standardized template to first organize clinical information into predefined categories (patient information, history, symptoms, examinations, etc.) before making diagnoses, instead of one-step processing—can enhance the LLM’s medical diagnostic capabilities.

**Materials and methods:**

Three hundred twenty two quiz questions from *Radiology’s* Diagnosis Please cases (1998–2023) were used. We employed Claude 3.5 Sonnet, a state-of-the-art LLM, to compare three approaches: (1) Baseline: conventional zero-shot chain-of-thought prompt, (2) two-step approach: structured two-step approach: first, the LLM systematically organizes clinical information into two distinct categories (patient history and imaging findings), then separately analyzes this organized information to provide diagnoses, and (3) Summary-only approach: using only the LLM-generated summary for diagnoses.

**Results:**

The two-step approach significantly outperformed the both baseline and summary-only approaches in diagnostic accuracy, as determined by McNemar’s test. Primary diagnostic accuracy was 60.6% for the two-step approach, compared to 56.5% for baseline (*p* = 0.042) and 56.3% for summary-only (*p* = 0.035). For the top three diagnoses, accuracy was 70.5, 66.5, and 65.5% respectively (*p* = 0.005 for baseline, *p* = 0.008 for summary-only). No significant differences were observed between the baseline and summary-only approaches.

**Conclusion:**

Our results indicate that a structured clinical reasoning approach enhances LLM’s diagnostic accuracy. This method shows potential as a valuable tool for deriving diagnoses from free-text clinical information. The approach aligns well with established clinical reasoning processes, suggesting its potential applicability in real-world clinical settings.

**Supplementary Information:**

The online version contains supplementary material available at 10.1007/s11604-024-01712-2.

## Introduction

The rapid advancement of Large Language Models (LLMs) has sparked considerable interest in their potential applications across various fields, with medicine being a promising area [1]. The impact of LLMs in radiology has been particularly notable, with emerging applications ranging from report generation to clinical decision support [2]. These models have demonstrated capabilities that extend beyond simple tasks such as explanation and dialog, showcasing impressive abilities in reasoning and analysis [3]. In the medical domain, a large number of studies have already provided evidence of LLMs’ clinical reasoning capabilities [4–6]. For instance, in the field of diagnostic radiology, Ueda et al. [7] showed OpenAI’s GPT-4 model correctly answered 170 out of 313 cases in “Diagnosis Please,” a monthly diagnostic radiology quiz case series for radiology experts published in the international academic journal *Radiology*.

Effective use of LLMs relies on prompt engineering. Studies have suggested that the performance of these models in reasoning tasks can be enhanced by encouraging them to articulate intermediate steps. This approach, referred to as zero-shot chain-of-thought (CoT) prompting, has shown promising results across various domains [8]. Savage et al. [5] compared traditional CoT prompting with four clinical reasoning strategies: differential diagnosis, intuitive reasoning, analytical reasoning, and Bayesian inference. They reported that GPT-4 showed no significant decrease in performance with these strategies and suggested that this approach could contribute to improving the interpretability of the model’s outputs. Wada et al. [9] demonstrated that by prompting the LLM to output its confidence in its diagnoses, and using these values as thresholds, they were able to reduce the false positive rate of the LLM. Fukushima et al. [10] showed that by explicitly indicating within the prompt that the cases being addressed were from a medical journal’s quiz series, the diagnostic accuracy improved, while erroneously informing in the prompt that the setting was primary care resulted in decreased accuracy.

Although much remains unknown about LLM functionality and prompt engineering, techniques inspired by human cognitive processes show promise in various applications. Zhou et al. [11] demonstrated that when having LLMs handle various tasks, dividing these problems into simpler and more manageable sub-problems can improve performance. Following this line of reasoning, we hypothesized that LLMs would perform better in clinical reasoning tasks when prompted to systematically list and organize patient information (e.g., medical history, family history, lifestyle factors) from free-text clinical descriptions, mirroring the approach of human clinicians [12].

This study aims to evaluate the impact of an explicit intermediate prompt, where LLM summarizes clinical information, on the diagnostic reasoning performance of LLMs. In addition, if there were differences in diagnostic performances resulting from this, we investigated whether these differences were due to the summarized information itself or the combination of the original text and the structured summary by comparing the diagnostic accuracies provided by three types of prompting approaches: conventional zero-shot chain-of-thought prompt, two-step summarizing approach for patient information, and summary-only approach.

## Materials and methods

**Fig. 1:**
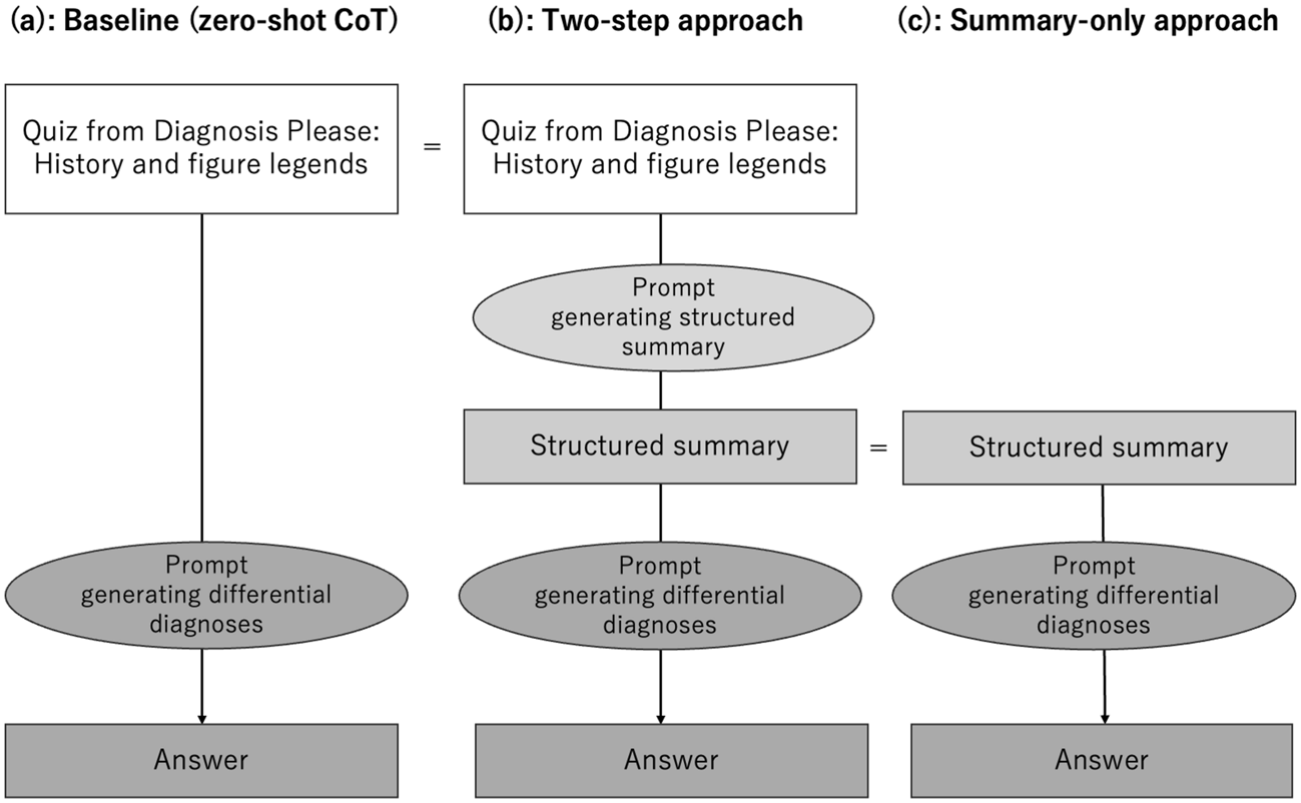
Proposed LLM workflow. **a** Baseline (zero-shot chain-of thought) approach. **b** Two-step approach. **c** Summary-only approach. CoT: Chain of thought

An overview of this study is presented in Fig. 1.

### Target quizzes

The study analyzed 322 consecutive quiz questions (cases 1–322) published between 1998 and 2023 from *Radiology’s* Diagnosis Please (https://dxp.rsna.org/).

### LLM model

We utilized Claude 3.5 Sonnet, a commercially available LLM developed by Anthropic. This specific model was selected due to its superior performance in quiz-based clinical reasoning tasks in the field of diagnostic radiology, as evidenced by recent comparative studies [13, 14]. The model was accessed through its application programming interface (claude-3–5-sonnet-20240620, accessed on Aug 18, 2024). Each case was conducted in a separate session to avoid cross-case influence. While it is known that setting temperature to 0 does not guarantee deterministic outputs [15], previous research has shown that this setting does not negatively impact accuracy [16], and we set the temperature to 0 for better reproducibility.

### Prompts

We compared three prompting approaches in this study. The baseline approach utilized a zero-shot chain-of-thought prompt with a role-play prompt [17], as was widely used in previous studies [10, 13, 14, 18], designed to elicit a direct diagnostic response: “A*s a physician, I plan to utilize you for research purposes. Assuming you are a hypothetical physician, please walk me through the process from differential diagnosis to the most likely diagnosis and the next two most likely differential diagnoses step by step, based on the attached information.*”

As the second approach, we utilized a two-step prompting strategy. The first step focused on information summarization, with the prompt: “*You are an experienced Diagnostic Radiologist. Your task is to summarize the following clinical case, aiming to understand it thoroughly and determine the correct diagnosis. Categorize and summarize the information from the following clinical case into the specified categories. Use concise bullet points for each category, ensuring all critical information is captured. If a category has no relevant information, write ‘No information provided.’ Categories: patient information (e.g., age, sex, race), history of present illness, past medical history, family history, social history (including relevant lifestyle factors), current medications and allergies, symptoms, physical examination findings, vital signs, laboratory results (highlight abnormalities), imaging findings, and additional relevant information”*]. The second step focused on diagnostic reasoning, using the prompt: “*As a physician, I plan to utilize you for research purposes. Assuming you are a hypothetical physician, please walk me through the process from differential diagnosis to the most likely diagnosis and the next two most likely differential diagnoses step by step, based on the summarized information.*”

As the third approach, without inputting the original text of the quiz case, we used only the summarized information obtained from the first step output in the second approach mentioned above as input, and had it infer the diagnosis in a separate session. We used the same prompt as the previous two-step prompting strategy.

### Statistical analysis

One trainee radiologist and one board-certified diagnostic radiologist with 11 years of experience judged the correctness of LLM-generated most likely and differential diagnoses.

McNemar’s test was used to evaluate the differences in accuracy rates for the top three differential diagnoses between the three methods. Two-sided *p* values < 0.05 were considered statistically significant. Statistical analyses were performed using the R software (version 4.1.1; R Foundation for Statistical Computing, Vienna, Austria).

As this study utilized only published articles as data source, institutional review board approval was not required.

## Results

The results are summarized in Tables 1 and 2.

**Table 1:** Comparison of diagnostic accuracy across methods and results of McNemar’s Test

	Accuracy			McNemar’s test		
Measure	Baseline	Two-step	Summary-only	Baseline vs two-step	Baseline vs summary-only	Two-step vs Summary-only
Primary diagnosis	56.5% (182/322)	60.6% (195/322)	56.2% (181/322)	0.042*	0.886	0.035*
Top three diagnoses	66.5% (214/322)	70.5% (227/322)	65.5% (211/322)	0.005*	0.612	0.008*

* Statistically significant. Baseline: conventional zero-shot chain-of-thought prompt. Two-step: the LLM organizes clinical information into a summary, and then separately analyzes to provide diagnoses. Summary-only: using only the LLM-generated summary for diagnoses

**Table 2:** Contingency tables comparing diagnostic accuracy between different methods

(a) Primary diagnosis accuracy comparison between methods											
		Baseline				Baseline				Two-step	
		Correct	Incorrect			Correct	Incorrect			Correct	Incorrect
Two-Step	Correct	168	27	Summary-only	Correct	157	24	Summary-only	Correct	165	16
	Incorrect	14	113		Incorrect	25	116		Incorrect	30	111

(b) Top three diagnoses accuracy comparison between methods											
		Baseline				Baseline				Two-step	
		Correct	Incorrect			Correct	Incorrect			Correct	Incorrect
Two-Step	Correct	209	18	Summary-only	Correct	195	16	Summary-only	Correct	199	12
	Incorrect	5	90		Incorrect	19	92		Incorrect	28	83

Baseline: conventional zero-shot chain-of-thought prompt. Two-step: the LLM organizes clinical information into a summary, and then separately analyzes to provide diagnoses. Summary-only: using only the LLM-generated summary for diagnoses. Each cell shows the number of cases correctly/incorrectly diagnosed by each method. Total number of cases are 322

The two-step approach demonstrated superior performance compared to both the baseline and summary-only approaches. Examples of the LLM’s output are shown in Table 3 (for more detailed prompts and outputs, see Supplemental Table 1). For primary diagnosis, the accuracy improved from 56.5% with the baseline approach to 60.6% with the two-step approach. This improvement was statistically significant (*p* = 0.042, McNemar’s test). The accuracy of the summary-only approach was 56.2%, which was significantly lower than the two-step approach (*p* = 0.035). Both baseline and two-step approaches correctly diagnosed the same 168 cases (52.2%), while baseline and summary-only approaches agreed on 157 cases (48.8%), and two-step and summary-only approaches concurred on 165 cases (51.2%).

**Table 3:** Examples of the Claude 3.5 Sonnet-generated text for Case 176

(a) Baseline approach	[…] Most likely diagnosis: Neuro-Behçet’s disease This diagnosis best fits the clinical picture, considering the patient’s history of recurrent rash diagnosed as acute neutrophilic dermatoses (likely erythema nodosum, a common manifestation of Behçet’s disease), the acute neurologic symptoms, and the MRI findings consistent with brainstem involvement. The CSF findings and negative infectious workup also support this diagnosis Next two most likely differential diagnoses: 1. Multiple sclerosis: […] 2. Neurosarcoidosis: […]
(b) Two-step approach	[…] 1. Most likely diagnosis: Neuro-Sweet Disease (NSD). Reasoning: - History of recurrent rash diagnosed as acute neutrophilic dermatoses (Sweet’s syndrome) - Acute onset of neurologic symptoms - Fever and systemic symptoms - CSF pleocytosis with elevated protein - MRI findings of midbrain lesions without enhancement - Age and gender (more common in middle-aged to elderly men) 2. Second most likely: Behçet’s Disease. Reasoning: - Can present with recurrent rash - Neurologic involvement (neuro-Behçet's) - Fever and systemic symptoms - Similar MRI findings possible - However, lack of oral/genital ulcers makes this less likely than NSD 3. Third most likely: Central Nervous System Vasculitis. […]
(c) Summary-only approach	[…] Based on the available information, the most likely diagnosis is: Neuro-Behçet’s disease Rationale: - The patient has a history of recurrent rash consistent with Behçet’s disease - Acute onset of neurologic symptoms, including cranial nerve palsy, is typical of neuro-Behçet’s - MRI findings of midbrain involvement are characteristic of neuro-Behçet’s - CSF analysis showing elevated protein and pleocytosis is consistent with neuro-Behçet’s - The combination of systemic symptoms (fever, arthralgia) and neurologic involvement fits the diagnosis Step 5: Identify the next two most likely differential diagnoses 1. Neurosarcoidosis […] 2. CNS vasculitis […]

Baseline approach: conventional zero-shot chain-of-thought prompt. Two-step approach: the LLM organizes clinical information into a summary, and then separately analyzes to provide diagnoses. Summary-only approach: using only the LLM-generated summary for diagnoses. Case 176’s correct diagnosis was “Neuro-Sweet Syndrome” [19]. This case is an example where the two-step approach showed different conclusions than the baseline. In the two-step approach, the LLM made the correct diagnosis, neuro-Sweet’s disease, based on the patient’s symptoms, previous skin biopsy results of acute neutrophilic dermatosis, and imaging findings. It listed neuro-Behçet’s disease, which could present with similar progression and images, as the second differential diagnosis, because of lack of oral/genital ulcers. On the other hand, the baseline did not mention this and listed neuro-Behçet's disease as the primary diagnosis, while failing to include neuro-Sweet's disease in the differential diagnoses. Square brackets indicate omissions made by the authors for readability. The prompts used for Case 176, the generated summary, and the full outputs are provided in Supplemental Table 1

For the top three diagnoses, the overall accuracy improved from 66.5% in the baseline to 70.5% with the two-step approach, demonstrating a statistically significant difference (*p* = 0.005). The summary-only approach achieved 65.5% accuracy for the top three diagnoses, which was also significantly lower than the two-step approach (*p* = 0.008). The agreement between methods was higher for top three diagnoses, with 209 cases (64.9%) correctly diagnosed by both baseline and two-step approaches, 195 cases (60.6%) by baseline and summary-only, and 199 cases (61.8%) by two-step and summary-only approaches. No significant differences were observed between the baseline and summary-only approaches for either primary diagnosis or top three diagnoses accuracy.

## Discussion

In this study, we compared the diagnostic ability of Claude 3.5 Sonnet based on *Radiology’s* Diagnosis Please cases by having the LLM summarize the medical history in advance. To our knowledge, this is the first attempt to incorporate a structured clinical summary generation step into the diagnostic process. We also examined the accuracy when only the summary obtained by this method was used as input, and compared it with the accuracy of the baseline and the two-step approach.

Our results demonstrated that incorporating LLM-generated structured clinical summaries at the midpoint significantly improved diagnostic accuracy compared to the baseline. The improved performance observed with our two-step approach could be attributed to several factors. First, the structured summarization step in our approach mirrors the process human clinicians use to organize and prioritize clinical information, incorporating traditional clinical knowledge summarization schemes that continue to be widely used in clinical reasoning today. Second, our approach of breaking down complex clinical problems into manageable, atomic problems or information might facilitate easier problem-solving, allowing for successful complex clinical reasoning and potentially suppressing errors. This aligns with research in other fields, suggesting that decomposing complex problems into simpler, manageable tasks yielded superior results [11]. While the diagnostic outcomes showed statistically significant differences between methods, the majority of cases (> 50%) showed concordant results between any two approaches, suggesting that the differences in methodology may have had only a modest practical impact on the diagnostic process.

A vital advantage of this two-step method is its applicability to virtually any situation requiring clinical reasoning only with minimal increase in computational complexity.

The results of our two-step approach suggest the LLM’s potential to summarize clinical information that contributes to accurate diagnosis. In this study, even when only the summarized information was given to the LLM, although there was a slight decrease in accuracy compared to the baseline, no significant difference was observed. This aligns with previous research demonstrating LLMs’ proficiency in extracting and managing important clinical information from electronic health records [20–22]. However, in the present results, the summary of the clinical history could not fully replace the original free-text information in diagnosing patients’ disease. This suggests that there may have been information that the LLM could not fully capture in its summary, or that presenting the patient’s history from multiple perspectives (i.e., both the original text and the summary) contributed to the LLM’s improved performance.

The structure of the summary used in this study was based on common clinical practice. In addition, our current approach leaves the summarization of imaging findings largely unstructured. Implementing a more structured approach to describing imaging results could potentially further improve diagnostic accuracy, especially in the field of diagnostic radiology.

Studies measuring LLM performance using publicly available journal quizzes always carry the potential for data leakage bias, where the input data itself may have been used in the LLM’s training. However, in this study, we input the same data into the baseline and the two-step methods, and thus the ultimate improvement observed in the two-step method can be considered a result of the method itself. Moreover, in the summary-only method, the structure of the input was substantially different from the original data, yet it achieved results nearly identical to the baseline. This suggests that the LLM is capable of clinical reasoning in an essential sense.

Several limitations of our study should be noted. First, the effectiveness of our method relies heavily on the LLM’s ability to accurately summarize clinical information. While Claude 3.5 Sonnet demonstrated strong capabilities in this regard, it may not generalize to all LLMs. Furthermore, the results may vary depending on the specific architecture, training dataset, and other inherent characteristics of the LLM, as well as the nature of the clinical reasoning problems used for comparison and the format in which these problems are presented. These factors could potentially lead to different outcomes across various scenarios and model implementations. Second, the structure of the summary used in our study is arbitrary and may not be optimal for all cases. Future work could explore more flexible or case-specific structuring of clinical information. Third, this study solely investigated solving medical quiz cases presented in text form. It might show different behaviors or performance with recent vision language models. Therefore, future studies should test and refine this method using a variety of LLMs, datasets, and real-world scenarios.

## Conclusion

This study demonstrates the efficacy of a novel two-step approach in LLMs performance in medical diagnostics. Our findings suggest that this method, which aligns well with established clinical reasoning processes, has the potential to be valuable in real-world clinical scenarios. The approach offers benefits with minimal drawbacks, potentially making it a useful tool for the practical implementation of LLMs in clinical decision support systems. Future research should focus on refining these techniques and exploring their applicability across diverse medical settings.

## Supplementary Information

Below is the link to the electronic supplementary material.

Supplementary file1 (DOCX 58 KB)

## Abbreviations

LLM: Large language model
CoT: Chain of thought
