# Supplementary material for: Structured clinical reasoning prompt enhances LLM’s diagnostic capabilities in diagnosis please quiz cases
Source: Jpn J Radiol. 2024 Dec 3;43(4):586–92. doi: 10.1007/s11604-024-01712-2 (PMC11953165; doi:10.1007/s11604-024-01712-2)
Supplement: Supplementary file 1 — Supplementary file1 (DOCX 58 KB) [file 11604_2024_1712_MOESM1_ESM.docx]

| **Supplementary Table 1: Prompts, Generated Summary, and Outputs for Case 176** |
| --- |
| **(a) Prompt utilized in the baseline (zero-shot chain-of-thought) method** # Request As a physician, I plan to utilize you for research purposes. Assuming you are a hypothetical physician, please walk me through the process from differential diagnosis to the most likely diagnosis and the next two most likely differential diagnoses step by step, based on the attached information.  # Clinical case History: A 75-year-old man presented with a 1-week history of fever, chills, arthralgia, and a sense of generalized weakness. He also had double vision for 1 day associated with one episode of urinary incontinence and an erythematous rash, which started on his neck and moved to his extremities. His medical history included a recurrent rash on his extremities since 2004. A prior skin biopsy revealed acute neutrophilic dermatoses. At physical examination, red nontender plaques were seen on the patient’s neck and extremities. Neurologic examination revealed evidence of mild disorientation. The results of motor, sensory, and coordination examinations were normal. The patient had horizontal diplopia on lateral gaze that was secondary to left abducens nerve palsy. The patient’s vital signs and systemic examination results were unremarkable. Cerebrospinal fluid analysis revealed an elevated concentration of proteins and pleocytosis; however, the glucose level was normal. Cerebrospinal fluid work-up was negative for herpes simplex virus with polymerase chain reaction testing, negative for Cryptococcus neoformans with antigen testing, and negative for bacteria with Gram stain and cultures. Initial computed tomography (CT) of the head revealed generalized atrophy without any acute changes. Subsequently, magnetic resonance (MR) imaging of the brain was performed ( Figs 1 – 4 ).  Imaging findings: Figure 1: Axial T2-weighted MR image (repetition time msec/echo time msec, 4754/101) shows patchy high signal intensity (arrow) in the midbrain. Figure 2: Axial FLAIR MR image (repetition time msec/echo time msec/inversion time msec, 9000/104/2500) shows patchy high signal intensity (arrow) in the midbrain. Figure 3: Axial diffusion-weighted MR image (6200/79) shows restricted diffusion (arrow) in the midbrain in the region of T2-weighted and FLAIR high signal intensity. Figure 4: Axial T1-weighted magnetization-prepared rapid acquisition gradient echo gadolinium-enhanced (10 mL of gadobutrol, 604.72 mg of gadolinium per milliliter, Gadovist; Bayer Healthcare Pharmaceuticals, Wayne, NJ) MR image (1900/3.37/1100) does not show any contrast enhancement in the areas of abnormal T2 and FLAIR signal intensity. |
| **(b) Prompt generating structured summary** # Role You are an experienced Diagnostic Radiologist. Your task is to summarize the following clinical case, aiming to understand it thoroughly and determine the correct diagnosis.  # Request Categorize and summarize the information from the following clinical case into the specified categories. Use concise bullet points for each category, ensuring all critical information is captured. If a category has no relevant information, write "No information provided."  Categories:  Patient Information (e.g., age, sex, race) History of Present Illness Past Medical History Family History Social History (including relevant lifestyle factors) Current Medications and Allergies Symptoms Physical Examination Findings Vital Signs Laboratory Results (highlight abnormalities) Imaging Findings Additional Relevant Information |
| **(c) Generated structured summary** Here's a categorized summary of the clinical case:  Patient Information: • 75-year-old man  History of Present Illness: • 1-week history of fever, chills, arthralgia, and generalized weakness • Double vision for 1 day • One episode of urinary incontinence • Erythematous rash starting on neck, moving to extremities  Past Medical History: • Recurrent rash on extremities since 2004 • Prior skin biopsy showed acute neutrophilic dermatoses  Family History: • No information provided  Social History: • No information provided  Current Medications and Allergies: • No information provided  Symptoms: • Fever, chills • Arthralgia • Generalized weakness • Double vision • Urinary incontinence • Erythematous rash  Physical Examination Findings: • Red nontender plaques on neck and extremities • Mild disorientation • Normal motor, sensory, and coordination examinations • Left abducens nerve palsy causing horizontal diplopia on lateral gaze  Vital Signs: • Unremarkable  Laboratory Results: • Cerebrospinal fluid analysis: - Elevated protein concentration - Pleocytosis - Normal glucose level - Negative for herpes simplex virus (PCR) - Negative for Cryptococcus neoformans (antigen testing) - Negative for bacteria (Gram stain and cultures)  Imaging Findings: • Initial CT head: Generalized atrophy, no acute changes • MRI brain: - T2-weighted and FLAIR: Patchy high signal intensity in midbrain - Diffusion-weighted: Restricted diffusion in midbrain - T1-weighted post-contrast: No enhancement in areas of abnormal T2 and FLAIR signal intensity  Additional Relevant Information: • No information provided |
| **(d) Output of the baseline (zero-shot chain-of-thought) approach** As a hypothetical physician, I'll walk you through the process of developing a differential diagnosis and identifying the most likely diagnosis based on the provided information.  **Step 1: Analyze the presenting symptoms and history** - 75-year-old man - 1-week history of fever, chills, arthralgia, and generalized weakness - Double vision for 1 day - One episode of urinary incontinence - Erythematous rash starting on neck and moving to extremities - History of recurrent rash on extremities since 2004 - Previous skin biopsy showing acute neutrophilic dermatoses  **Step 2: Consider the physical examination findings** - Red nontender plaques on neck and extremities - Mild disorientation - Left abducens nerve palsy causing horizontal diplopia  **Step 3: Review the diagnostic test results** - CSF analysis: elevated proteins and pleocytosis, normal glucose - Negative CSF tests for HSV, Cryptococcus, and bacteria - CT head: generalized atrophy, no acute changes - MRI brain: patchy high signal intensity in midbrain on T2 and FLAIR, restricted diffusion, no contrast enhancement  **Step 4: Develop a differential diagnosis** Based on the clinical presentation, history, and imaging findings, the differential diagnosis includes:  1. Neuro-Behçet's disease 2. Multiple sclerosis 3. Neurosarcoidosis 4. Acute disseminated encephalomyelitis (ADEM) 5. Central nervous system vasculitis  **Step 5: Analyze the evidence for each diagnosis**  1. Neuro-Behçet's disease: - Recurrent rash consistent with acute neutrophilic dermatoses - Neurological symptoms (diplopia, urinary incontinence) - MRI findings consistent with brainstem involvement - CSF pleocytosis  2. Multiple sclerosis: - Neurological symptoms - MRI lesions - CSF pleocytosis - However, the acute onset and systemic symptoms are less typical  3. Neurosarcoidosis: - Can cause neurological symptoms and rash - MRI lesions - CSF pleocytosis - However, lack of typical systemic involvement (e.g., pulmonary)  4. ADEM: - Acute onset - Neurological symptoms - MRI lesions - However, typically affects younger individuals and often follows an infection or vaccination  5. CNS vasculitis: - Can cause neurological symptoms and systemic inflammation - MRI lesions - However, lack of typical vascular distribution of lesions  **Step 6: Determine the most likely diagnosis and next two most likely differential diagnoses**  Most likely diagnosis: Neuro-Behçet's disease This diagnosis best fits the clinical picture, considering the patient's history of recurrent rash diagnosed as acute neutrophilic dermatoses (likely erythema nodosum, a common manifestation of Behçet's disease), the acute neurological symptoms, and the MRI findings consistent with brainstem involvement. The CSF findings and negative infectious workup also support this diagnosis.  Next two most likely differential diagnoses:  1. Multiple sclerosis: While less likely due to the acute onset and systemic symptoms, it remains a possibility given the neurological symptoms and MRI findings.  2. Neurosarcoidosis: This condition can present with similar neurological symptoms and MRI findings, but the lack of typical systemic involvement makes it less likely than Neuro-Behçet's disease.  In conclusion, based on the available information, Neuro-Behçet's disease is the most likely diagnosis, with multiple sclerosis and neurosarcoidosis as the next two most likely differential diagnoses. Further testing, such as HLA-B51 genetic testing and a thorough rheumatological workup, would be recommended to confirm the diagnosis of Neuro-Behçet's disease. |
| **(e) Output of the two-step approach** Certainly, I'd be happy to walk you through the diagnostic process as a hypothetical physician. Let's approach this step-by-step:  **Step 1: Analyze the key symptoms and findings**  The patient presents with: - Acute onset of fever, chills, arthralgia, and weakness - Neurological symptoms (double vision, urinary incontinence, mild disorientation) - Recurrent rash with a history of acute neutrophilic dermatoses - CSF showing pleocytosis and elevated protein - MRI findings of midbrain lesions with restricted diffusion  **Step 2: Formulate a differential diagnosis**  Given these findings, we need to consider conditions that can affect both the skin and central nervous system, particularly those that can cause midbrain lesions. The main categories to consider are:  1. Autoimmune/inflammatory conditions 2. Infectious diseases 3. Vascular disorders 4. Neoplastic processes  **Step 3: Evaluate each potential diagnosis**  1. Most likely diagnosis: Neuro-Sweet Disease (NSD)  Reasoning: - History of recurrent rash diagnosed as acute neutrophilic dermatoses (Sweet's syndrome) - Acute onset of neurological symptoms - Fever and systemic symptoms - CSF pleocytosis with elevated protein - MRI findings of midbrain lesions without enhancement - Age and gender (more common in middle-aged to elderly men)  2. Second most likely: Behçet's Disease  Reasoning: - Can present with recurrent rash - Neurological involvement (neuro-Behçet's) - Fever and systemic symptoms - Similar MRI findings possible - However, lack of oral/genital ulcers makes this less likely than NSD  3. Third most likely: Central Nervous System Vasculitis  Reasoning: - Can cause multifocal neurological symptoms - May present with systemic symptoms like fever - Can have associated skin manifestations - MRI findings could be consistent - However, the specific history of neutrophilic dermatoses makes this less likely than NSD  **Step 4: Conclusion**  The most likely diagnosis is Neuro-Sweet Disease (NSD), a rare neurological manifestation of Sweet's syndrome. This diagnosis best fits the patient's history of neutrophilic dermatoses, the acute onset of neurological symptoms, fever, and the MRI findings. The lack of contrast enhancement on MRI is also more consistent with NSD than with infectious or neoplastic processes.  To confirm this diagnosis, additional steps would include: 1. Skin biopsy of current lesions to confirm neutrophilic infiltration 2. HLA typing (HLA-B54 is associated with NSD) 3. Exclusion of other causes through further serological tests (e.g., ANA, ANCA for vasculitis) 4. Trial of corticosteroid therapy, as NSD typically responds well to this treatment  While Behçet's Disease and CNS Vasculitis remain in the differential, the specific history of neutrophilic dermatoses and the overall clinical picture make NSD the most likely diagnosis in this case. |
| **(f) Output of the summary-only approach** Thank you for providing this detailed clinical summary. As a hypothetical physician, I'll walk you through the process of developing a differential diagnosis and identifying the most likely diagnosis, along with the next two most likely differential diagnoses.  **Step 1: Analyze key symptoms and findings** The patient presents with: - Acute onset of neurological symptoms (double vision, urinary incontinence, mild disorientation) - Systemic symptoms (fever, chills, arthralgia, generalized weakness) - Recurrent rash with a history of acute neutrophilic dermatoses - CSF analysis showing elevated protein and pleocytosis - MRI findings of patchy high signal intensity and restricted diffusion in the midbrain  **Step 2: Develop a broad differential diagnosis** Given the combination of neurological, systemic, and dermatological symptoms, we should consider: 1. Neuro-Behçet's disease 2. Neurosarcoidosis 3. Central nervous system (CNS) vasculitis 4. Infectious encephalitis (viral, bacterial, or fungal) 5. Autoimmune encephalitis 6. Multiple sclerosis 7. Acute disseminated encephalomyelitis (ADEM)  **Step 3: Narrow down the differential diagnosis** We can narrow our focus based on the specific findings: - The history of recurrent rash and prior diagnosis of acute neutrophilic dermatoses - The acute onset of neurological symptoms - The MRI findings specific to the midbrain - The CSF analysis results  **Step 4: Identify the most likely diagnosis** Based on the available information, the most likely diagnosis is:  Neuro-Behçet's disease  Rationale: - The patient has a history of recurrent rash consistent with Behçet's disease - Acute onset of neurological symptoms, including cranial nerve palsy, is typical of neuro-Behçet's - MRI findings of midbrain involvement are characteristic of neuro-Behçet's - CSF analysis showing elevated protein and pleocytosis is consistent with neuro-Behçet's - The combination of systemic symptoms (fever, arthralgia) and neurological involvement fits the diagnosis  **Step 5: Identify the next two most likely differential diagnoses**  1. Neurosarcoidosis  Rationale: - Can present with multisystem involvement, including skin and nervous system - Can cause cranial nerve palsies and meningeal inflammation - MRI findings can be similar to those seen in this case - CSF analysis can show elevated protein and pleocytosis  2. CNS vasculitis  Rationale: - Can present with acute neurological symptoms and systemic inflammation - MRI findings can show patchy areas of involvement - CSF analysis can reveal elevated protein and pleocytosis - The patient's age puts him at risk for various forms of vasculitis  These diagnoses would require further investigation to rule out or confirm. Additional tests such as serum ACE levels, chest imaging, and potentially a brain biopsy might be necessary to differentiate between these conditions definitively. |

| **Supplementary Table 2: Clinical Case, Imaging Findings, Generated Summary, and Outputs for Case 85, 146, 166 and 203** |  |
| --- | --- |
| **Case 85 [1]** History: A 45-year-old woman with a medical history of constipation and irritable bowel syndrome presented with cramps in the left lower abdomen. She had experienced these symptoms for about 2 months. The symptoms were accompanied by decreased appetite and weight loss of 6 kg over 2 months. For a few weeks, she experienced fevers up to 39°C and cold shivers. Physical examination revealed tenderness in the left lower quadrant of the abdomen, without a palpable mass. A mass anterior to the rectum was felt with digital rectal examination. Laboratory findings included an elevated C-reactive protein level of 160 mg/L (normal range,   10 mg/L), an elevated erythrocyte sedimentation rate of 108 mm/h (normal range, 20 mm/h), and a high white blood cell count of 17.9   109/L (normal range, [4.3–10.0]  109/L). Contrast material–enhanced abdominal computed tomography (CT) was performed.  Imaging findings: Sequential transverse abdominal and pelvic CT images obtained after administration of oral, rectal, and intravenous contrast material. (a) Abdominal CT image shows hydronephrosis (arrow) of the left kidney. (b) Pelvic CT image shows an abscess (arrow). B   bladder, U   uterus. (c) Pelvic CT image shows an IUD (short arrow) and infiltrated perirectal fat (long arrow). B   bladder, R   rectum. (d) Pelvic CT image shows perirectal fluid (arrow). T   tampon. |  |
| **Generated structured summary** Here's a categorized summary of the clinical case:  Patient Information:  • 45-year-old woman  History of Present Illness:  • Cramps in left lower abdomen for about 2 months  • Decreased appetite  • Weight loss of 6 kg over 2 months  • Fevers up to 39°C and cold shivers for a few weeks  Past Medical History:  • Constipation  • Irritable bowel syndrome  Family History:  • No information provided  Social History:  • No information provided  Current Medications and Allergies:  • No information provided  Symptoms:  • Abdominal cramps  • Decreased appetite  • Weight loss  • Fever  • Cold shivers  Physical Examination Findings:  • Tenderness in left lower quadrant of abdomen  • No palpable abdominal mass  • Mass anterior to rectum felt on digital rectal examination  Vital Signs:  • No information provided  Laboratory Results:  • C-reactive protein: 160 mg/L (elevated; normal <10 mg/L)  • Erythrocyte sedimentation rate: 108 mm/h (elevated; normal <20 mm/h)  • White blood cell count: 17.9 × 10^9/L (elevated; normal 4.3–10.0 × 10^9/L)  Imaging Findings:  • Abdominal CT: Hydronephrosis of left kidney  • Pelvic CT:  - Abscess  - IUD present  - Infiltrated perirectal fat  - Perirectal fluid  - Presence of tampon  Additional Relevant Information:  • Contrast-enhanced abdominal CT performed with oral, rectal, and intravenous contrast material |  |
| **Output of the baseline (zero-shot chain-of-thought) approach**  As a hypothetical physician, I'll walk you through the process of developing a differential diagnosis based on the provided information:  **Step 1: Analyze the presenting symptoms and history**  - 45-year-old woman  - Left lower abdominal cramps for 2 months  - Decreased appetite and weight loss (6 kg in 2 months)  - Fevers up to 39°C and cold shivers for a few weeks  - History of constipation and irritable bowel syndrome  **Step 2: Consider the physical examination findings**  - Tenderness in the left lower quadrant of the abdomen  - Mass anterior to the rectum felt on digital rectal examination  **Step 3: Review laboratory results**  - Elevated C-reactive protein (160 mg/L)  - Elevated erythrocyte sedimentation rate (108 mm/h)  - High white blood cell count (17.9 × 10^9/L)  **Step 4: Analyze imaging findings**  - Left kidney hydronephrosis  - Pelvic abscess  - IUD present  - Infiltrated perirectal fat  - Perirectal fluid  **Step 5: Develop a differential diagnosis**  Based on the clinical presentation, laboratory findings, and imaging results, the top three differential diagnoses are:  1. Diverticulitis with abscess formation (Most likely diagnosis)  2. Pelvic inflammatory disease (PID) with tubo-ovarian abscess  3. Colorectal cancer with abscess formation  **Step 6: Reasoning for the most likely diagnosis**  - Diverticulitis with abscess formation  - The patient's age (45) and history of constipation increase the risk of diverticulitis  - Left lower quadrant pain is typical for sigmoid diverticulitis  - Fever, elevated inflammatory markers, and leukocytosis suggest an infectious process  - CT findings of pelvic abscess, infiltrated perirectal fat, and perirectal fluid are consistent with complicated diverticulitis  - The presence of hydronephrosis suggests possible ureteral involvement, which can occur in severe diverticulitis  **Step 7: Reasoning for the second most likely diagnosis**  - Pelvic inflammatory disease (PID) with tubo-ovarian abscess  - The presence of an IUD increases the risk of PID  - Pelvic pain, fever, and elevated inflammatory markers are consistent with PID  - CT findings of a pelvic abscess could represent a tubo-ovarian abscess  **Step 8: Reasoning for the third most likely diagnosis**  - Colorectal cancer with abscess formation  - The patient's age puts her at risk for colorectal cancer  - Weight loss and decreased appetite could be associated with malignancy  - The palpable mass on rectal examination could represent a tumor  - Abscess formation can occur as a complication of colorectal cancer  In conclusion, while all three diagnoses are possible, the clinical presentation and imaging findings are most consistent with complicated diverticulitis. Further evaluation, such as colonoscopy after resolution of the acute inflammation, may be necessary to definitively rule out colorectal cancer. |  |
| **Output of the two-step approach**  Certainly, I'd be happy to walk you through the diagnostic process as a hypothetical physician. Let's approach this step-by-step:  **Step 1: Analyze the key symptoms and findings**  The patient presents with:  - Left lower abdominal cramps  - Weight loss  - Fever  - Abdominal tenderness  - Rectal mass  - Elevated inflammatory markers  - CT findings of abscess, perirectal inflammation, and left hydronephrosis  **Step 2: Formulate a differential diagnosis**  Given these findings, we should consider:  1. Diverticulitis with abscess formation  2. Inflammatory bowel disease (e.g., Crohn's disease)  3. Colorectal cancer  4. Pelvic inflammatory disease (PID)  5. Tubo-ovarian abscess  **Step 3: Evaluate each diagnosis**  1. Diverticulitis with abscess:  - Consistent with left-sided abdominal pain, fever, and abscess on CT  - Can explain the inflammatory markers and weight loss  - Common in this age group  2. Inflammatory bowel disease:  - Can cause abdominal pain, weight loss, and fever  - May present with abscesses and fistulas  - Less likely given the acute presentation and lack of chronic symptoms  3. Colorectal cancer:  - Can cause weight loss and changes in bowel habits  - May present with abscess if perforated  - Less likely given the acute inflammatory picture  4. Pelvic inflammatory disease:  - Consistent with pelvic pain, fever, and abscess  - Presence of IUD increases risk  - Typically presents with more upper genital tract symptoms  5. Tubo-ovarian abscess:  - Can cause lower abdominal pain and fever  - Often associated with PID  - Less likely given the perirectal involvement  **Step 4: Determine the most likely diagnosis**  The most likely diagnosis is Diverticulitis with abscess formation.  Reasoning:  - Left-sided abdominal pain is typical for diverticulitis  - Presence of fever and elevated inflammatory markers suggest an infectious/inflammatory process  - CT findings of abscess and perirectal inflammation are consistent with complicated diverticulitis  - The patient's age is appropriate for diverticulitis  - Weight loss can occur due to decreased appetite and systemic inflammation  **Step 5: Identify the next two most likely differential diagnoses**  1. Pelvic Inflammatory Disease (PID):  - The presence of an IUD increases the risk of PID  - Can present with lower abdominal pain, fever, and abscess formation  - However, the perirectal involvement is less typical  2. Inflammatory Bowel Disease (specifically Crohn's disease):  - Can cause abdominal pain, weight loss, and fever  - May present with abscesses and fistulas  - The acute presentation makes this less likely than diverticulitis, but it remains a possibility  These diagnoses would require further investigation, including colonoscopy, to definitively rule out or confirm. |  |
| **Output of the summary-only approach** As a hypothetical physician, I'll walk you through the process of developing a differential diagnosis and identifying the most likely diagnoses based on the provided information.  **Step 1: Analyze the key symptoms and findings**  - Abdominal cramps (left lower quadrant)  - Weight loss  - Fever  - Elevated inflammatory markers (CRP, ESR, WBC)  - Pelvic abscess on CT  - Hydronephrosis of left kidney  - Presence of IUD and tampon  **Step 2: Generate a broad differential diagnosis**  Consider conditions that could explain the combination of abdominal pain, systemic symptoms, and pelvic abscess:  1. Pelvic Inflammatory Disease (PID)  2. Diverticulitis with abscess formation  3. Inflammatory Bowel Disease (IBD) - Crohn's disease  4. Tubo-ovarian abscess  5. Appendicitis with abscess formation  6. Colorectal cancer with abscess  7. Pelvic actinomycosis  **Step 3: Evaluate each diagnosis based on the available information**  **Step 4: Determine the most likely diagnosis and next two most likely differentials**  Most likely diagnosis: Pelvic Inflammatory Disease (PID) with abscess formation  Reasoning:  - Presence of IUD, which is a risk factor for PID  - Pelvic abscess on CT  - Fever and elevated inflammatory markers  - Abdominal pain in the left lower quadrant  - Hydronephrosis, which can occur due to inflammation and abscess formation  Next two most likely differential diagnoses:  1. Diverticulitis with abscess formation  Reasoning:  - Left lower quadrant pain  - Abscess on CT  - Fever and elevated inflammatory markers  - More common in older patients, but possible at 45 years old  - History of constipation, which is a risk factor for diverticulitis  2. Inflammatory Bowel Disease (IBD) - Crohn's disease  Reasoning:  - Abdominal pain  - Weight loss  - Fever  - Elevated inflammatory markers  - Abscess formation is common in Crohn's disease  - History of IBS, which can sometimes be misdiagnosed IBD  These diagnoses best fit the clinical picture presented, with PID being the most likely due to the presence of an IUD and the typical presentation. However, further investigation would be necessary to confirm the diagnosis and rule out other possibilities. |  |
| **Case 146 [2]** History: A 27-year-old gravida 1, para 1 woman who had a history of pelvic inflammatory disease presented to the emergency department with lower abdominal pain, nausea, vomiting, and chills. She began menstruating the night before presentation and reported an acute exacerbation of her chronic abdominal pain, which typically worsened with her menses. Her surgical history included one caesarean section and laparoscopic excision of a tubo-ovarian abscess. She was afebrile. Physical examination revealed mild diffuse abdominal tenderness and tenderness of the cervix. Laboratory findings revealed a white blood cell count of 17 000 cells per cubic millimeter. (A normal white blood cell count is between 4800 and 11 000 cells per cubic millimeter.) Ultrasonography (US) of the pelvis was performed. A computed tomographic (CT) examination of the abdomen and pelvis was subsequently performed after intravenous administration of 125 mL of iohexol (Omnipaque; Amersham Health, Princeton, NJ). We acquired 5-mm axial sections and performed 3-mm coronal reconstruction.  Imaging findings: Figure 1: Sagittal transabdominal US image of the left adnexa reveals a multicystic mass (arrows) and a normal left ovary ( ). Figure 2: Axial transabdominal Doppler US image of the right lower quadrant of the abdomen shows the multicystic mass, with no color flow to the multiple septa (arrow). Figure 3: Oral (20 mL iohexol diluted with 430 mL of water) and intravenous contrast-enhanced axial CT image of the pelvis reveals multiple septa (arrowheads) within the fluid-attenuation mass. Figure 4: Oral and intravenous contrast-enhanced coronal CT image of the abdomen and pelvis shows multiple septa (arrows) in the fluidattenuation mass, which extends from the pelvis to the right upper quadrant of the abdomen and displaces multiple small-bowel loops to the left. No scalloping of the liver or spleen is shown. |  |
| **Generated structured summary** Here's a categorized summary of the clinical case:  Patient Information:  • 27-year-old female  • Gravida 1, para 1  History of Present Illness:  • Presented to emergency department with lower abdominal pain, nausea, vomiting, and chills  • Menstruation started the night before presentation  • Acute exacerbation of chronic abdominal pain, typically worsening with menses  Past Medical History:  • History of pelvic inflammatory disease  • One caesarean section  • Laparoscopic excision of a tubo-ovarian abscess  Family History:  • No information provided  Social History:  • No information provided  Current Medications and Allergies:  • No information provided  Symptoms:  • Lower abdominal pain  • Nausea  • Vomiting  • Chills  Physical Examination Findings:  • Mild diffuse abdominal tenderness  • Tenderness of the cervix  Vital Signs:  • Afebrile  Laboratory Results:  • White blood cell count: 17,000 cells per cubic millimeter (elevated; normal range 4,800-11,000)  Imaging Findings:  • Ultrasonography (US):  - Multicystic mass in left adnexa  - Normal left ovary  - No color flow to multiple septa on Doppler US  • CT scan (with oral and intravenous contrast):  - Fluid-attenuation mass with multiple septa  - Mass extends from pelvis to right upper quadrant of abdomen  - Displacement of multiple small-bowel loops to the left  - No scalloping of liver or spleen  Additional Relevant Information:  • CT performed with 125 mL of iohexol (Omnipaque)  • CT protocol: 5-mm axial sections with 3-mm coronal reconstruction |  |
| **Output of the baseline (zero-shot chain-of-thought) approach** As a hypothetical physician, I'll walk you through the process of developing a differential diagnosis based on the provided information, then narrow it down to the most likely diagnosis and the next two most likely differential diagnoses.  **Step 1: Analyze the patient's history and symptoms**  - 27-year-old woman with a history of pelvic inflammatory disease  - Previous caesarean section and laparoscopic excision of tubo-ovarian abscess  - Presenting with lower abdominal pain, nausea, vomiting, and chills  - Pain exacerbates with menses  - Afebrile, but with mild diffuse abdominal tenderness and cervical tenderness  - Elevated white blood cell count (17,000 cells/mm³)  **Step 2: Review imaging findings**  - Multicystic mass in the left adnexa  - Normal left ovary visible  - No color flow to the multiple septa on Doppler US  - Fluid-attenuation mass with multiple septa extending from pelvis to right upper quadrant  - Displacement of small bowel loops  - No scalloping of liver or spleen  **Step 3: Develop initial differential diagnosis**  Based on the history, symptoms, and imaging findings, the initial differential diagnosis includes:  1. Endometriosis with endometrioma  2. Ovarian cyst or cystadenoma  3. Tubo-ovarian abscess  4. Hydrosalpinx  5. Peritoneal inclusion cyst  **Step 4: Narrow down the differential diagnosis**  Considering the imaging findings of a large multicystic mass with multiple septa, no color flow, and extension from the pelvis to the right upper quadrant, we can narrow down our differential:  Most likely diagnosis: Peritoneal inclusion cyst (PIC)  Reasoning:  - Multicystic appearance with multiple septa  - Extension from pelvis to upper abdomen  - History of pelvic inflammatory disease and previous surgery  - Pain exacerbation with menses  - No color flow on Doppler US  - Normal left ovary visible separately from the mass  Next two most likely differential diagnoses:  1. Endometriosis with endometrioma  Reasoning:  - History of cyclic pain worsening with menses  - Multicystic appearance possible in advanced endometriosis  - Previous pelvic inflammatory disease and surgery are risk factors  2. Hydrosalpinx  Reasoning:  - History of pelvic inflammatory disease  - Cystic appearance on imaging  - Can cause chronic pelvic pain  **Step 5: Conclusion**  The most likely diagnosis is a peritoneal inclusion cyst, given the imaging findings and patient history. This diagnosis explains the large multicystic mass, lack of blood flow, and the patient's history of pelvic inflammatory disease and previous surgery. The cyclic nature of the pain and its exacerbation with menses are also consistent with this diagnosis.  Endometriosis with endometrioma and hydrosalpinx remain in the differential diagnosis but are less likely given the specific imaging findings and the extent of the cystic mass. Further evaluation, such as MRI or laparoscopy, might be necessary for definitive diagnosis and treatment planning. |  |
| **Output of the two-step approach** Certainly, I'd be happy to walk you through the diagnostic process as a hypothetical physician. Let's approach this step-by-step:  **Step 1: Analyze the key clinical features**  - 27-year-old female with history of pelvic inflammatory disease  - Acute exacerbation of chronic abdominal pain, worsening with menses  - Lower abdominal pain, nausea, vomiting, and chills  - Elevated white blood cell count  - Multicystic mass extending from pelvis to right upper quadrant  **Step 2: Formulate a broad differential diagnosis**  Given these features, we should consider:  1. Endometriosis  2. Ovarian cyst or tumor  3. Pelvic inflammatory disease (PID) recurrence  4. Tubo-ovarian abscess  5. Peritoneal inclusion cyst (PIC)  6. Appendicitis  7. Ectopic pregnancy  **Step 3: Narrow down the differential based on imaging findings**  The imaging findings of a multicystic mass with multiple septa, extending from the pelvis to the right upper quadrant, narrow our differential to:  1. Peritoneal inclusion cyst (PIC)  2. Ovarian cyst or tumor  3. Endometriosis with endometriomas  **Step 4: Consider the patient's history and clinical presentation**  The patient's history of PID and previous tubo-ovarian abscess, along with the cyclical nature of pain exacerbation, further refines our differential.  **Step 5: Arrive at the most likely diagnosis**  Based on all the information, the most likely diagnosis is:  Peritoneal Inclusion Cyst (PIC)  Reasoning:  - History of pelvic inflammatory disease and previous surgery  - Multicystic mass with multiple septa on imaging  - Lack of blood flow within the septa on Doppler US  - Extension of the mass from pelvis to upper abdomen  - Cyclical exacerbation of pain with menses  **Step 6: Identify the next two most likely differential diagnoses**  1. Endometriosis with endometriomas  Reasoning:  - Cyclical pain exacerbation with menses  - History of pelvic pathology  - Multicystic appearance on imaging (could represent multiple endometriomas)  2. Complex ovarian cyst or tumor  Reasoning:  - Multicystic appearance on imaging  - Abdominal pain and mass  - Can occur in women of reproductive age  In conclusion, while Peritoneal Inclusion Cyst is the most likely diagnosis given the patient's history and imaging findings, endometriosis and complex ovarian cyst/tumor remain important considerations in the differential diagnosis. Further management would likely include gynecological consultation and possibly laparoscopy for definitive diagnosis and treatment. |  |
| **Output of the summary-only approach** As a hypothetical physician, I'll walk you through the process of developing a differential diagnosis and identifying the most likely diagnosis based on the provided information.  **Step 1: Analyze the key symptoms and findings**  - Lower abdominal pain, exacerbated by menses  - Nausea, vomiting, and chills  - History of pelvic inflammatory disease and tubo-ovarian abscess  - Elevated white blood cell count  - Multicystic mass in left adnexa on ultrasound  - Large fluid-attenuation mass with multiple septa on CT scan  **Step 2: Generate a broad differential diagnosis**  Based on the patient's symptoms, history, and imaging findings, we should consider:  1. Endometriosis  2. Ovarian cyst or cystadenoma  3. Tubo-ovarian abscess  4. Pelvic inflammatory disease  5. Ectopic pregnancy  6. Appendicitis  7. Ovarian torsion  8. Uterine fibroids  9. Pelvic congestion syndrome  10. Malignant ovarian tumor  **Step 3: Narrow down the differential diagnosis**  We can eliminate some options based on the available information:  - Ectopic pregnancy is less likely due to the patient's menstruation  - Appendicitis is less likely due to the left-sided mass  - Ovarian torsion is less likely due to the normal left ovary on ultrasound  - Uterine fibroids are less likely due to the adnexal location of the mass  **Step 4: Analyze the most likely diagnosis**  The most likely diagnosis is Endometriosis, specifically an Endometrioma (chocolate cyst).  Reasoning:  1. Chronic abdominal pain worsening with menses  2. History of pelvic inflammatory disease, which can be associated with endometriosis  3. Multicystic mass in the adnexa  4. No color flow to multiple septa on Doppler US, consistent with an endometrioma  5. Large fluid-attenuation mass with multiple septa on CT, typical of endometriomas  6. Patient's age and reproductive history are consistent with endometriosis  **Step 5: Consider the next two most likely differential diagnoses**  1. Ovarian Cystadenoma:  - Can present as a large, multicystic mass  - May cause abdominal pain and discomfort  - However, less likely to be associated with cyclic pain during menses  2. Tubo-ovarian Abscess:  - Patient's history of pelvic inflammatory disease and previous tubo-ovarian abscess  - Elevated white blood cell count  - Presence of chills  - However, the patient is afebrile, and the imaging findings are more consistent with an endometrioma  In conclusion, while endometriosis (endometrioma) is the most likely diagnosis, ovarian cystadenoma and tubo-ovarian abscess should be considered as alternative possibilities. Further evaluation, such as CA-125 levels, MRI, or laparoscopy, may be necessary to confirm the diagnosis and guide treatment. |  |
| **Case 166 [3]** History: A 54-year-old man had a history of hereditary nonpolyposis colorectal cancer. Colon cancer was initially diagnosed in 2001, and the patient underwent right colectomy. In 2002, the patient developed duodenal cancer, and he underwent a Whipple procedure. A second colon cancer was discovered in 2004, and the patient underwent completion colectomy. Surveillance body computed tomography (CT) was performed in 2007 and revealed a new right hilar mass. Additional imaging included fluorine 18 ( 18F) fluorodeoxyglucose (FDG) combined positron emission tomography (PET) and CT ( Figs 1 , 2 ) and follow-up intravenous contrast material–enhanced(90 mL of iohexol 300, Omnipaque; GE Healthcare, Fairfield, Conn) chest CT ( Fig 3 )..  Imaging findings: Figure 1: Anterior maximum intensity projection 18 F FDG PET/CT image. Three regions of abnormal FDG uptake can be seen on this image, including a large hypermetabolic mass in the right hilum, a small focus located more superiorly and to the left (arrow), and a faint focus in the left hilum (arrowhead).  Figure 2: (a, c) Unenhanced axial CT and (b, d) 18 F FDG PET images obtained just above and just below the carina. CT images were obtained with soft-tissue window settings. (a, c) There is a hypermetabolic lesion in the region of the left pulmonary artery and AP window (arrow). (b, d) The large right hilar mass and a faint focus in the left hilum (arrow) can be seen.  Figure 3: (a, b) Axial contrast-enhanced CT images obtained just above and just below the carina with soft-tissue window settings. (a) The lesion seen at PET/CT can be more clearly seen to cause a partial filling defect within the left pulmonary artery (arrow). (b) The large right hilar mass is well delineated (arrow), and an eccentric soft-tissue defect is present in the left lower lobe pulmonary artery (arrowhead). |  |
| **Generated structured summary** Here's a categorized summary of the clinical case:  Patient Information:  • 54-year-old man  History of Present Illness:  • Hereditary nonpolyposis colorectal cancer  • Colon cancer diagnosed in 2001, underwent right colectomy  • Duodenal cancer in 2002, underwent Whipple procedure  • Second colon cancer in 2004, underwent completion colectomy  • New right hilar mass discovered on surveillance CT in 2007  Past Medical History:  • Multiple cancers as described in History of Present Illness  Family History:  • Hereditary nonpolyposis colorectal cancer (suggesting family history)  Social History:  No information provided  Current Medications and Allergies:  No information provided  Symptoms:  No information provided  Physical Examination Findings:  No information provided  Vital Signs:  No information provided  Laboratory Results:  No information provided  Imaging Findings:  • 18F FDG PET/CT:  - Large hypermetabolic mass in the right hilum  - Small hypermetabolic focus superior and left to the main mass  - Faint hypermetabolic focus in the left hilum  • Contrast-enhanced CT chest:  - Partial filling defect within the left pulmonary artery  - Large right hilar mass well delineated  - Eccentric soft-tissue defect in the left lower lobe pulmonary artery  Additional Relevant Information:  • Patient underwent multiple imaging studies including:  - Surveillance body CT (2007)  - 18F FDG PET/CT  - Contrast-enhanced chest CT (90 mL of iohexol 300) |  |
| **Output of the baseline (zero-shot chain-of-thought) approach** As a hypothetical physician, I'll walk you through the process of developing a differential diagnosis and determining the most likely diagnosis based on the provided information.  **Step 1: Analyze the patient's history**  - 54-year-old male  - History of hereditary nonpolyposis colorectal cancer (HNPCC)  - Multiple cancers: colon cancer (2001), duodenal cancer (2002), second colon cancer (2004)  - Multiple surgeries: right colectomy, Whipple procedure, completion colectomy  - New right hilar mass discovered in 2007  **Step 2: Review imaging findings**  - PET/CT shows:  1. Large hypermetabolic mass in the right hilum  2. Small focus superiorly and to the left  3. Faint focus in the left hilum  - Contrast-enhanced CT shows:  1. Partial filling defect within the left pulmonary artery  2. Large right hilar mass  3. Eccentric soft-tissue defect in the left lower lobe pulmonary artery  **Step 3: Develop initial differential diagnosis**  1. Metastatic cancer (from previous colorectal or duodenal cancers)  2. Primary lung cancer  3. Pulmonary embolism  4. Lymphoma  5. Sarcoidosis  **Step 4: Analyze and prioritize differential diagnoses**  1. Metastatic cancer:  - Most likely diagnosis given the patient's extensive cancer history  - Multiple lesions in different locations support metastatic spread  - HNPCC increases risk of multiple primary cancers and metastases  2. Primary lung cancer:  - Second most likely diagnosis  - Multiple lesions could represent primary lung cancer with metastases  - Smoking history not provided, but still a possibility given age and gender  **3. Pulmonary embolism:**  - Third most likely diagnosis  - Filling defects in pulmonary arteries are consistent with pulmonary emboli  - Cancer patients have an increased risk of thromboembolism  **Step 5: Conclusion**  Most likely diagnosis: Metastatic cancer (likely from previous colorectal or duodenal primary)  Next two most likely differential diagnoses:  1. Primary lung cancer with metastases  2. Pulmonary embolism (potentially coexisting with cancer)  To confirm the diagnosis, further steps would include:  1. Biopsy of the right hilar mass  2. Complete blood count and tumor markers  3. Pulmonary function tests  4. Consideration of anticoagulation therapy if pulmonary embolism is suspected  This approach takes into account the patient's extensive cancer history, imaging findings, and the likelihood of various conditions based on the presented information. |  |
| **Output of the two-step approach** Certainly, I'd be happy to walk you through the diagnostic process as a hypothetical physician. Let's approach this step-by-step:  **Step 1: Analyze the key information**  The patient is a 54-year-old man with:  - History of hereditary nonpolyposis colorectal cancer (HNPCC)  - Multiple previous cancers: colon (2001, 2004), duodenal (2002)  - New right hilar mass discovered on surveillance CT in 2007  - PET/CT showing hypermetabolic lesions in right hilum, left pulmonary artery region, and left hilum  - CT showing filling defects in pulmonary arteries  **Step 2: Formulate a differential diagnosis**  Given the patient's history and imaging findings, we should consider:  1. Metastatic cancer (from previous colorectal or duodenal primaries)  2. New primary lung cancer  3. Pulmonary embolism  4. Sarcoma  5. Lymphoma  **Step 3: Analyze each possibility**  1. Metastatic cancer:  - Highly likely given the patient's extensive cancer history  - Multiple lesions in different lung areas support this  - HNPCC patients are prone to multiple primary cancers  2. New primary lung cancer:  - Possible, as HNPCC patients have an increased risk of various cancers  - Multiple lesions make this less likely as a sole explanation  3. Pulmonary embolism:  - Filling defects in pulmonary arteries are suggestive  - However, hypermetabolic activity on PET is atypical for simple PE  4. Sarcoma:  - Less likely, but possible given the soft tissue masses  - Uncommon in this setting  5. Lymphoma:  - Possible, but less likely given the patient's history and presentation  **Step 4: Determine the most likely diagnosis**  The most likely diagnosis is Metastatic Cancer, specifically metastases from the patient's previous colorectal or duodenal cancers.  Reasoning:  - Patient's history of multiple cancers related to HNPCC  - Multiple hypermetabolic lesions on PET/CT suggestive of metastases  - Filling defects in pulmonary arteries likely represent tumor thrombi rather than simple pulmonary emboli  **Step 5: Identify the next two most likely differential diagnoses**  1. New Primary Lung Cancer with Metastases  Reasoning:  - HNPCC patients are at risk for multiple primary cancers  - The right hilar mass could be a primary lung cancer with metastases to other lung areas  2. Pulmonary Tumor Thrombotic Microangiopathy (PTTM)  Reasoning:  - Explains the filling defects in pulmonary arteries  - Associated with underlying malignancy, fitting the patient's history  - Can cause multiple PET-avid lesions due to tumor emboli  These diagnoses would require further investigation, potentially including biopsy of the right hilar mass and/or one of the other lesions to confirm the diagnosis and guide treatment. |  |
| **Output of the summary-only approach** As a hypothetical physician, I'll walk you through the process of developing a differential diagnosis based on the provided information:  **Step 1: Analyze the patient's history and risk factors**  - 54-year-old male with hereditary nonpolyposis colorectal cancer (HNPCC)  - Multiple previous cancers: colon cancer (2001, 2004), duodenal cancer (2002)  - Underwent multiple surgeries for these cancers  **Step 2: Consider the new findings**  - Right hilar mass discovered on surveillance CT in 2007  - PET/CT shows hypermetabolic masses in right and left hilum  - CT chest shows partial filling defect in left pulmonary artery and eccentric soft-tissue defect in left lower lobe pulmonary artery  **Step 3: Develop a differential diagnosis**  Given the patient's history and new findings, the top three differential diagnoses are:  1. Most likely diagnosis: Metastatic cancer (likely of colorectal origin)  Reasoning:  - Patient's history of multiple cancers associated with HNPCC  - New hypermetabolic masses in both hila  - HNPCC patients are at increased risk for extracolonic cancers  2. Second most likely diagnosis: Primary lung cancer  Reasoning:  - Hilar masses can be indicative of primary lung cancer  - Smoking history not provided, but lung cancer can occur in non-smokers  - Multiple hypermetabolic foci in both hila  3. Third most likely diagnosis: Pulmonary embolism with tumor thrombus  Reasoning:  - Partial filling defect in left pulmonary artery  - Eccentric soft-tissue defect in left lower lobe pulmonary artery  - History of multiple cancers increases risk of thromboembolism  **Step 4: Consider next steps**  - Biopsy of the right hilar mass to confirm diagnosis and determine origin  - Further imaging studies to evaluate for other metastatic sites  - Consult oncology for management planning  - Consider genetic counseling and testing for family members due to HNPCC  This approach takes into account the patient's complex medical history, current imaging findings, and the likelihood of various diagnoses based on the available information. Further diagnostic tests and specialist consultations would be necessary to confirm the diagnosis and guide treatment. |  |
| **Case 203 [4]** History: A 77-year-old woman presented with pain in the right elbow of several years duration. There was no history of trauma, immunocompromise, or known malignancy. Blood chemistry tests revealed an alkaline phosphatase level of 93 U/L (1.6 mkat/L) (30–120 U/L [0.5–2.0 mkat/L]), a sodium level of 139 mmol/L (135–147 mmol/L), a potassium level of 4.4 mmol/L (3.4–5.0 mmol/L), a calcium level of 2.38 mmol/L (2.2–2.55 mmol/L), a phosphate level of 1.81 mmol/L (0.85–1.5 mmol/L), a C-reactive protein level of less than 5 mg/L (47.6 nmol/L), and an erythrocyte sedimentation rate of 41 mm/h (0–15 mm/h). Hematologic findings were normal. Imaging included plain film radiography, computed tomography (CT), and magnetic resonance (MR) imaging of the right humerus (Figs 1–3). Bone biopsies were performed 3 months and 4 years after the patient’s initial presentation.  Imaging findings: Figure 1: Anteroposterior radiograph of the right humerus shows welldefined small lucencies in the distal humerus, with thinning and loss of bone cortex. There is no visible tumor matrix calcification or periosteal reaction.  Figure 2: (a) Unenhanced CT image of the right humerus shows punched-out defects in the cortex of the distal right humerus but no soft-tissue mass. (b) Coronal CT reconstruction shows absence of periosteal reaction or soft-tissue mass. (c) Three-dimensional CT reconstruction shows large circumscribed lucencies in the distal humerus.  Figure 3: (a) Coronal T2-weighted short inversion time inversion-recovery (2000/60/150) 1.5-T MR image shows elongated vascular structures adjacent to humeral diaphysis with slow flow. (b) Coronal T1-weighted spectral presaturation with inversion recovery gadolinium-enhanced (400/12) 1.5-T MR image shows enhancement of vascular structures filling the lytic lesions. (c) Axial T1-weighted spectral presaturation with inversion recovery gadolinium-enhanced (479/14) 1.5-T MR image shows enhancement in veins of the upper arm and humerus. | |
| **Generated structured summary** Here's a categorized summary of the clinical case:  Patient Information:  • 77-year-old woman  History of Present Illness:  • Pain in the right elbow for several years  • No history of trauma  Past Medical History:  • No known malignancy  • Not immunocompromised  Family History:  • No information provided  Social History:  • No information provided  Current Medications and Allergies:  • No information provided  Symptoms:  • Pain in the right elbow  Physical Examination Findings:  • No information provided  Vital Signs:  • No information provided  Laboratory Results:  • Alkaline phosphatase: 93 U/L (normal)  • Sodium: 139 mmol/L (normal)  • Potassium: 4.4 mmol/L (normal)  • Calcium: 2.38 mmol/L (normal)  • Phosphate: 1.81 mmol/L (elevated)  • C-reactive protein: <5 mg/L (normal)  • Erythrocyte sedimentation rate: 41 mm/h (elevated)  • Hematologic findings: normal  Imaging Findings:  • X-ray: Well-defined small lucencies in distal humerus, thinning and loss of bone cortex  • CT: Punched-out defects in cortex of distal right humerus, no soft-tissue mass or periosteal reaction  • MRI: Elongated vascular structures adjacent to humeral diaphysis with slow flow, enhancement of vascular structures filling lytic lesions  Additional Relevant Information:  • Bone biopsies performed 3 months and 4 years after initial presentation  • No visible tumor matrix calcification on X-ray | |
| **Output of the baseline (zero-shot chain-of-thought) approach** As a hypothetical physician, I'll walk you through the process of developing a differential diagnosis and narrowing it down to the most likely diagnosis based on the information provided.  **Step 1: Initial Differential Diagnosis**  Given the patient's age, chronic elbow pain, and imaging findings, we should consider the following initial differential diagnoses:  1. Metastatic disease  2. Multiple myeloma  3. Primary bone tumor (benign or malignant)  4. Vascular malformation  5. Infection (osteomyelitis)  6. Rheumatoid arthritis  **Step 2: Analyzing Available Information**  Clinical presentation:  - 77-year-old woman  - Right elbow pain for several years  - No history of trauma, immunocompromise, or known malignancy  Laboratory findings:  - Most blood tests are within normal limits  - Slightly elevated phosphate level  - Elevated erythrocyte sedimentation rate (ESR)  Imaging findings:  - Well-defined lucencies in the distal humerus  - Thinning and loss of bone cortex  - No visible tumor matrix calcification or periosteal reaction  - No soft-tissue mass  - Elongated vascular structures adjacent to humeral diaphysis  - Enhancement of vascular structures filling the lytic lesions  **Step 3: Narrowing Down the Differential**  Based on the available information, we can start eliminating some diagnoses:  1. Infection (osteomyelitis) is less likely due to the chronic nature of symptoms and normal C-reactive protein level.  2. Rheumatoid arthritis is less likely due to the unilateral presentation and lack of typical joint involvement.  3. Multiple myeloma is less likely due to normal calcium levels and the absence of other typical symptoms.  **Step 4: Most Likely Diagnosis**  The most likely diagnosis based on the clinical presentation and imaging findings is:  Intraosseous Vascular Malformation (Hemangioma)  Reasons:  - Well-defined lytic lesions without periosteal reaction  - Presence of elongated vascular structures on MRI  - Enhancement of vascular structures filling the lytic lesions  - Chronic nature of symptoms  - Absence of soft-tissue mass or aggressive bone destruction  **Step 5: Next Two Most Likely Differential Diagnoses**  1. Metastatic Disease  Reasons to consider:  - Patient's age  - Lytic bone lesions  - Elevated ESR  Reasons it's less likely:  - No known primary malignancy  - Lack of aggressive bone destruction or soft-tissue mass  - Normal alkaline phosphatase and calcium levels  2. Primary Bone Tumor (e.g., Giant Cell Tumor)  Reasons to consider:  - Lytic bone lesions  - Thinning of cortex  Reasons it's less likely:  - Patient's age (typically occurs in younger patients)  - Lack of periosteal reaction or soft-tissue mass  - Presence of vascular structures on MRI  In conclusion, while intraosseous vascular malformation appears to be the most likely diagnosis, further investigation, including the results of the bone biopsies mentioned, would be crucial to confirm the diagnosis and rule out other possibilities. | |
| **Output of the two-step approach** Certainly, I'd be happy to walk you through the diagnostic process as a hypothetical physician. Let's approach this step-by-step:  **Step 1: Analyze the key findings**  The most significant findings in this case are:  - 77-year-old woman with chronic right elbow pain  - No history of trauma or known malignancy  - Elevated phosphate and ESR  - Imaging showing lytic lesions in the distal humerus with associated vascular structures  **Step 2: Formulate a differential diagnosis**  Based on these findings, our initial differential diagnosis should include:  1. Primary bone tumor (benign or malignant)  2. Metastatic disease  3. Multiple myeloma  4. Infection (osteomyelitis)  5. Vascular malformation  6. Hyperparathyroidism  **Step 3: Evaluate each possibility**  1. Primary bone tumor: Possible, but the patient's age and the multilocular nature of the lesions make this less likely.  2. Metastatic disease: Possible, but there's no known primary malignancy.  3. Multiple myeloma: Age-appropriate, but normal calcium and lack of other typical findings make this less likely.  4. Infection: Chronic osteomyelitis could present similarly, but normal CRP and lack of systemic symptoms make this less likely.  5. Vascular malformation: The imaging findings strongly suggest this, particularly the MRI showing vascular structures.  6. Hyperparathyroidism: Could cause lytic lesions, but calcium levels are normal.  **Step 4: Determine the most likely diagnosis**  Given the imaging findings of lytic lesions with associated vascular structures, the lack of soft tissue mass, and the chronic nature of the symptoms, the most likely diagnosis is:  Primary diagnosis: Intraosseous Venous Malformation (previously known as intraosseous hemangioma)  **Step 5: Identify the next two most likely differential diagnoses**  Second most likely: Metastatic Disease  - Although there's no known primary malignancy, the patient's age and the presence of lytic lesions warrant consideration of this possibility.  Third most likely: Atypical presentation of Multiple Myeloma  - Given the patient's age and the presence of lytic bone lesions, this remains a possibility, although the normal calcium and lack of other typical findings make it less likely.  **Step 6: Recommend further actions**  To confirm the diagnosis and rule out other possibilities:  1. Biopsy results (already performed) should be reviewed  2. Consider a full body scan to rule out other lesions or primary malignancy  3. If not already done, perform serum protein electrophoresis to rule out multiple myeloma  4. Consider angiography for better characterization of the vascular malformation if treatment is being considered  This approach takes into account the clinical presentation, laboratory findings, and imaging results to arrive at the most likely diagnosis while keeping other important possibilities in mind. | |
| **Output of the summary-only approach** As a hypothetical physician, I'll walk you through the process of developing a differential diagnosis based on the provided information:  **Step 1: Analyze the key findings**  - 77-year-old woman with right elbow pain for several years  - No history of trauma or known malignancy  - Imaging shows lytic lesions in the distal humerus  - Elevated phosphate and ESR  - Normal alkaline phosphatase, calcium, and CRP  - MRI shows vascular structures adjacent to the humeral diaphysis  **Step 2: Generate a broad differential diagnosis**  Given the patient's age, chronic pain, and imaging findings, we should consider:  1. Primary bone tumors (benign or malignant)  2. Metastatic disease  3. Metabolic bone disorders  4. Inflammatory conditions  5. Vascular anomalies  **Step 3: Narrow down the differential based on specific findings**  - The presence of well-defined lytic lesions without periosteal reaction or soft tissue mass suggests a benign process.  - Normal alkaline phosphatase and calcium levels make metabolic bone disorders less likely.  - The vascular structures seen on MRI are a key finding.  **Step 4: Identify the most likely diagnosis**  Based on the imaging findings, especially the MRI showing vascular structures filling the lytic lesions, the most likely diagnosis is:  Intraosseous Hemangioma  **Step 5: Identify the next two most likely differential diagnoses**  1. Giant Cell Tumor of Bone  Rationale:  - Can occur in older adults  - Typically presents as a lytic lesion in long bones  - Can cause local pain  - Normal alkaline phosphatase  2. Multiple Myeloma  Rationale:  - More common in older adults  - Can present with lytic bone lesions  - Elevated ESR  - Normal calcium (although often elevated in multiple myeloma)  **Step 6: Explain why these are less likely than the primary diagnosis**  1. Giant Cell Tumor of Bone:  - Usually occurs in younger patients (20-40 years old)  - Typically affects the epiphyseal region of long bones  - Lacks the characteristic vascular findings seen on MRI  2. Multiple Myeloma:  - Usually presents with multiple lytic lesions throughout the skeleton  - Often associated with anemia and elevated calcium, which are not present in this case  - Lacks the vascular component seen on MRI  The presence of vascular structures filling the lytic lesions, as seen on MRI, strongly supports the diagnosis of intraosseous hemangioma over the other possibilities. The chronic nature of the symptoms, lack of aggressive features on imaging, and normal lab values (except for mildly elevated phosphate and ESR) also align more closely with a benign vascular tumor than with the other differential diagnoses. | |

Note. The correct diagnoses for these cases are: Case85: Pelvic Actinomycosis in Association with an Intrauterine Device, Case 146: Benign Multicystic Mesothelioma, Case 166: Metastatic Left Pulmonary Artery Sarcoma, and Case 203: Gorham Disease.

References

1. Lely RJ, Van Es HW. Case 85: Pelvic Actinomycosis in Association with an Intrauterine Device. Radiology. 2005;236:492–4.

2. Koo PJ, Wills JS. Case 146: Benign Multicystic Mesothelioma. Radiology. 2009;251:944–6.

3. Mittra ES, Iagaru AH, Leung AN. Case 166: Metastatic Left Pulmonary Artery Sarcoma. Radiology. 2011;258:645–8.

4. De Villiers JFK, Stevens WR. Case 203: Gorham Disease. Radiology. 2014;270:931–5.
